# Long-term management of recurrent biofilms in the tear trough regions following hyaluronic acid filler treatment

**DOI:** 10.1016/j.jdcr.2025.01.036

**Published:** 2025-03-04

**Authors:** Kaitlyn M. Enright, Lucie Khouri, Quynh Nguyen, Kiana Zanetti, Steven Bernstein, Andreas Nikolis

**Affiliations:** a Erevna Innovations Inc, Clinical Research Unit, Westmount, Quebec, Canada; b Département d’ophtalmologie, Université de Montréal, Montréal, Québec, Canada; c Victoria Park Medispa, Westmount, Québec, Canada; d Division of Plastic Surgery, McGill University, Montreal, Quebec, Canada

**Keywords:** adverse events, delayed complications, inflammatory reactions, periorbital injections, pharmacovigilance

## Introduction

Most adverse events associated with hyaluronic acid (HA) fillers are mild in severity, resolve spontaneously and promptly, and are associated with the injection technique rather than the product.^1^^,^^2^ However, serious adverse events [eg, biofilms, foreign body granulomas] can arise months to years following treatment. Serious adverse events are more likely to be associated with the product itself.^2^, ^3^, ^4^ Similarities between clinical manifestations of infections and immune reactions can make proper diagnosis and treatment difficult. We present a rare case of recurrent masses in the bilateral tear trough regions following HA filler injections.

## Case report

The patient was a 50-year-old female with a history of cigarette smoking, slightly raised cholesterol levels, and seasonal allergies often accompanied by periorbital swelling. She remarked that her periorbital allergy symptoms had essentially resolved following an aesthetic lower lid blepharoplasty in 2006.

Six months following the blepharoplasty, she noticed bilateral hollowing in the tear troughs. The operating surgeon was unable to propose a suitable solution. The patient consulted another surgeon who recommended a HA filler volumizing treatment. At that time, the patient was naïve to esthetic injections. The patient received a bilateral volume of 3.50 mL HA injections in the tear troughs and malar regions. Days after the injections, the patient observed edema near the injection sites. She also felt pressure and tingling in the areas. There was no follow-up with the injecting nurse. The tingling sensation subsided after several weeks, but the edema persisted.

**Fig 1:**
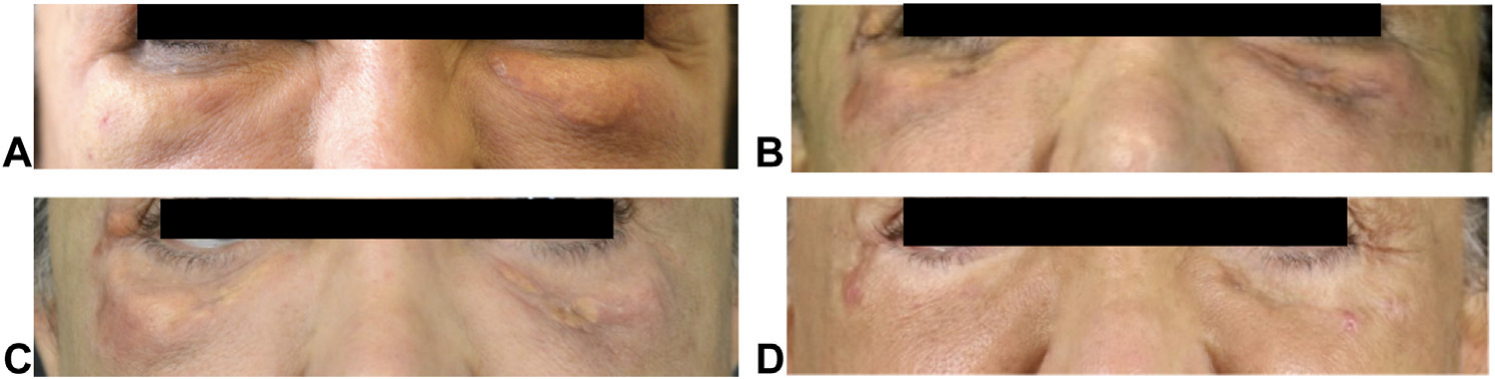
Recurrent masses in the bilateral tear troughs. **A,** Patient in 2016 upon presenting to the plastic surgeon. **B,** In July 2017 after the bilateral lower blepharoplasty. **C,** Appearance of new growths in September 2017. **D,** Patient in December 2020 following multiple excisions.

After 1 year of persistent edema, the patient consulted a dermatologist. The patient presented with firm, immobile, and painless masses (Fig 1) in the periorbital, sub-orbital and mid-cheek areas. The dermatologist attempted to dissolve the HA with intralesional hyaluronidase monthly for 6 months, but the edema persisted. For almost 4 years, the patient lived with abnormal masses increasing in size and number.

She consulted an oculoplastic surgeon who attempted treatment with intralesional cortisone and prescribed a 1-month supply of antibiotics. The patient received regular cortisone injections for almost 2 years. She remarked that the injections decreased the edema marginally. The clinician eventually discontinued this treatment as it caused the skin below the patient’s eyes to turn yellow. The oculoplastic surgeon consulted with her colleague, a plastic surgeon.

**Fig 2:**
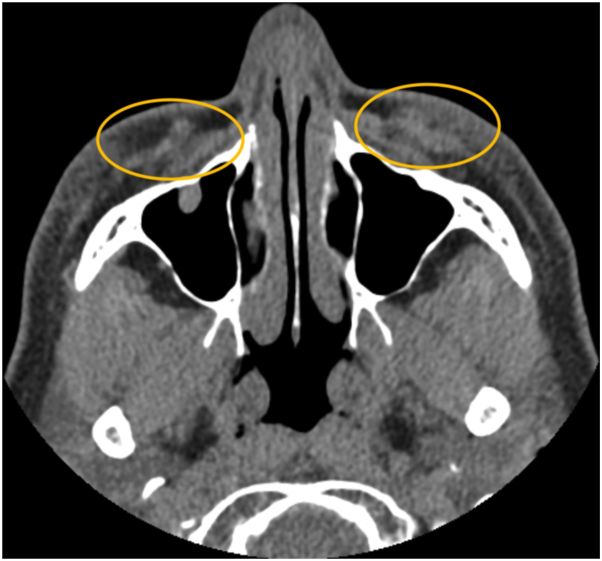
Masses in the bilateral tear troughs on computed tomography scan. Sample image from the patient’s computed tomography scan. Masses (*circled*) are integrated into the skin in the regions injected with hyaluronic acid filler.

As the edema and masses appeared years after the initial esthetic treatment, the plastic surgeon suspected the development of biofilms as a secondary effect of the original injections. He began a 3-month treatment regimen for the patient. He administered intralesional hyaluronidase 1500 units/mL × 4 (to pursue removal of remnant HA) and intralesional clindamycin for 5 days during the first month. During the second month, he administered intralesional hyaluronidase 1500 units/mL, 300 units per side. He prescribed a 7-day course of prednisone during the third month. While the appearance of the masses improved, some fullness persisted. Two months later, new masses appeared. The plastic surgeon sent the patient for a computerized tomography scan. The computerized tomography scan showed abnormal growths integrated into her skin (Fig 2).

**Fig 3:**
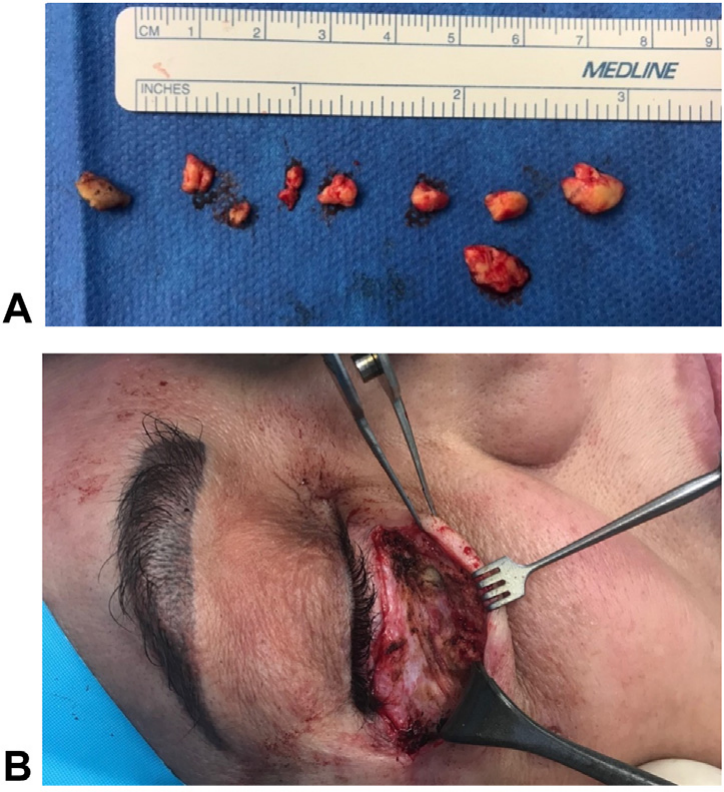
Periorbital mass excisions. **A,** Samples of masses excised with scale. **B,** The affected region.

In May 2017, the plastic surgeon performed a bilateral lower blepharoplasty to debulk the masses. He sent excisional biopsies to pathology (Fig 3; Table I).

**Table I:** Assessments conducted on the patient and/or her tissue samples and their corresponding results

Year	Assessment	Diagnosis and/or treatment
2007	Hollowing of the tear troughs assessed by a dermatologist	Attempted treatment with hyaluronic acid injections in the tear troughs
2008	Dermatologist assessed AEs caused by hyaluronic acid injections	Attempted treatment with hyaluronidase injections every mo for 6 mo
2013	Persisting masses assessed by an oculoplastic surgeon	Attempted treatment with 1 mo of antibiotics, 2 y of cortisone
2016	Persisting masses assessed by an oculoplastic surgeon	Attempted treatment with intralesional antibiotics and hyaluronidase injections
2017	Surgical pathology report after infraorbital malar and cheek exploration	Excisions on both lower lids demonstrated florid histiocytic aggregates with foreign body-type giant cell reaction
2018	Specimen report from excisional biopsies of the bilateral lower lids	Florid histiocytic aggregates with foreign body-type giant cell reaction
2020	Surgical pathology report after excisions on the right lower lid	Florid histiocytic aggregates with foreign body-type giant cell reaction

Subsequent treatment efforts focused on serially excising the recurrent masses and the damaged tissue from the HA injections. The plastic surgeon performed 3 more serial excisions 6 months later. In 2018, he performed 2 further excisions on the lower lid region. In 2019, he performed 2 bilateral excisions on the lower lids. In a final surgery in December 2020, he excised 2 masses and corrected an ectropion of the lower right eyelid. The surgeon sent masses from this surgery to pathology (Table I).

By 2020, the excisions significantly improved the patient's condition. New masses appeared less frequently and were smaller, not exceeding 3 mm × 5 mm. Between December 2020 and May 2021, the patient returned monthly for follow-up. She received monthly fat injections for aesthetic improvement. Her condition remained stable as of her last visit in May 2021. The patient passed away in 2023 due to complications from amyotrophic lateral sclerosis.

## Discussion

Granulomas and implant nodules may form with delayed onset as the muscles of the face redistribute ill-positioned filler particles.^5^^,^^6^ Nodules result from technical error and appear sooner than granulomas (1-2 months after injection), after initial swelling subsides. Nodules remain small, while granulomas grow and may extend into surrounding tissues.^6^ Histologically, granuloma particles are scattered. Nodule particles form aggregates. Given these clinical descriptions, some clinicians treated the masses as granulomas. However, granulomas are usually treated successfully with corticosteroids, which did not resolve the current case.^7^ Biofilm formation was initially ruled out, given the negative bacterial culture. However, given reports of biofilms providing false negatives on bacterial cultures, this option was again considered.^8^ Soft tissue fillers have been associated with biofilms. Suspected causes include non-sterile injection techniques that may have introduced bacteria into the injection sites via a contaminated needle.^9^ External triggers such as a recent viral infection or immunization may also reactivate an existing, dormant biofilm.^10^ Most cases of biofilms have been associated with the use of permanent fillers (eg polymethylmethacrylate), rather than HA, which is biocompatible and reversible.^9^ Unfortunately, no systemic treatment exists for this condition. Surgical techniques remain the most suitable management strategy, although they may lead to secondary tissue deficits. Biofilm formation may also be stimulated by the surgical procedure itself. Management efforts should include aesthetic improvement. For this patient, such efforts included fat grafting and corrective blepharoplasty. To avoid adverse events, injectors should use a slow, aseptic injection technique, deposit small aliquots of product, and undertreat critical areas. The key takeaway from this case is that reinjecting HA in a region with a known biofilm present is not advisable. For these patients, surgeons should advise against further HA filler injections and instead consider microfat grafting.

## Conflicts of interest

None disclosed.
